# Comparison of Single-Incision Scrotal Orchiopexy and Traditional Two-Incision Inguinal Orchiopexy for Primary Palpable Undescended Testis in Children: A Systematic Review and Meta-Analysis

**DOI:** 10.3389/fped.2022.805579

**Published:** 2022-03-15

**Authors:** Chengjun Yu, Yang Hu, Ling Wang, Lian Kang, Jie Zhao, Jiandong Lu, Tao Lin, Dawei He, Shengde Wu, Guanghui Wei

**Affiliations:** ^1^Department of Urology, Children's Hospital of Chongqing Medical University, Chongqing, China; ^2^National Clinical Research Center for Child Health and Disorders, Chongqing, China; ^3^Chongqing Key Laboratory of Children Urogenital Development and Tissue Engineering, Chongqing, China; ^4^Ministry of Education Key Laboratory of Child Development and Disorders, Chongqing, China; ^5^Chongqing Key Laboratory of Pediatrics, Chongqing, China; ^6^China International Science and Technology Cooperation Base of Child Development and Critical Disorders, Chongqing, China

**Keywords:** undescended testes, orchiopexy, single-incision, minimal invasive surgery, palpable undescended testes (PUDTs), single-incision scrotal orchiopexy (SISO), traditional two-incision inguinal orchiopexy (TTIO), children

## Abstract

**Purpose:**

To compare the safety, efficacy, and cosmetic results of single-incision scrotal orchiopexy (SISO) and traditional two-incision inguinal orchiopexy (TTIO) for primary palpable undescended testes (PUDTs) in children.

**Materials and Methods:**

A systematic literature search of all relevant studies published on PubMed, Embase, Medline, Cochrane Library, Web of Science database, and Wanfang data until July 2021 was conducted. The operative time, hospitalization duration, conversion rate, wound infection or dehiscence, scrotal hematoma or swelling, testicular atrophy, reascent, hernia or hydrocele, analgesics needs, and cosmetic results were compared between SISO and TTIO using the Mantel–Haenszel or inverse-variance method.

**Results:**

A total of 17 studies involving 2,627 children (1,362 SISOs and 1,265 TTIOs) were included in the final analysis. The conversion rate of SISO was 3.6%. The SISO approach had a statistically significant shorter operative time than the TTIO approach for PUDT (weighted mean difference−11.96, 95% confidence interval −14.33 to −9.59, I^2^ = 79%, *P* < 0.00001) and a shorter hospital stay (weighted mean difference−1.05, 95% confidence interval −2.07 to −0.03, *P* = 0.04). SISO needed fewer analgesics and had better cosmetic results than TTIO. SISO had a similar total, short-term, or long-term complication rate with TTIO.

**Conclusion:**

Compared with TTIO, SISO has the advantages of shorter operative time, shorter hospitalization duration, less postoperative pain, and better cosmetic appealing results. SISO is a safe, effective, promising, and potential minimal invasive surgical approach for PUDT. SISO is an alternative to TTIO in selected cryptorchid patients, especially for lower positioned ones.

**Systematic Review Registration:**

https://www.crd.york.ac.uk/PROSPERO/, identifier: CRD42021268562.

## Introduction

Cryptorchidism, or undescended testes (UDTs), is one of the most common congenital abnormalities in male neonates, with a prevalence of 1.0–4.6% in full-term boys and a higher incidence in preterm boys (1). Cryptorchidism is a well-known independent risk fact for infertility, testicular cancer, testicular torsion, and other disease (2). It is vital to correct the UDT at an early age to avoid subsequent testicular degeneration. Fortunately, ~80% of the UDT are palpable and located in the inguinal canal, external inguinal ring, or even upper scrotal (3), making the traditional two-incision inguinal orchiopexy (TTIO) the best surgical approach to correct cryptorchidism (4).

The TTIO has two incisions: one inguinal incision to open the external oblique fascia and inguinal canal to visualize the cord structure and dissect the processus vaginalis; another second scrotal incision to fix the descended testis within the scrotum (5, 6). It was believed that the TTIO was convenient and helpful for sufficient mobilization of the spermatic cord, separation and high ligation of the processus vaginalis or hernia sac to avoid subsequent hernia or hydrocele, and most importantly, to achieve adequate vessel length for cryptorchid testis to be placed in the scrotum without tension (7, 8).

In 1989, Bianchi and Squire proposed the single-incision scrotal orchiopexy (SISO) for palpable undescended testes (PUDTs) to reduce the potential morbidity and reach a goal for better cosmetic appearance (9). From then on, more and more authors pointed out that SISO had the advantages of shorter operative time and less pain; in the meanwhile, it also had considerable complication rates compared with TTIO (10–15). This trans-scrotal surgical technique could also be applied to secondary cryptorchidism, hydrocele, and even indirect hernias (16).

In 2016, Feng et al. conducted the first systematic review and meta-analysis to compare the SISO with TTIO strategy regarding operative time and complications (17). Although the evidence was still largely limited by the small sample size in their study, these several newly published randomized controlled studies would provide more evidence. Therefore, we updated this study and compared the operative time, hospitalization duration, patent processus vaginalis, short-term and long-term complications, analgesic needs, scarring, and conversions between SISO and TTIO for primary PUDT to provide strengthened evidence and to serve clinical decision and guideline making.

## Materials and Methods

### Search Strategy

This systematic review and meta-analysis were conducted in accordance with the Preferred Reporting Items for Systematic Reviews and Meta-analysis statement. The initial systematic literature retrieve was conducted on July 11, 2021, without the restriction of language and region. The searched databases were the following: PubMed, Medline, Embase, Cochrane Library, Web of Science Database, China National Knowledge Infrastructure, and WanFang Data. Using the Boolean approach, these databases were individually searched for the following key terms, mainly in titles, keywords, and abstracts: (cryptorchidism OR undescended test^*^ OR non-descended test^*^ OR non descended test^*^) AND (orchidopexy OR scrotal OR inguinal OR single incision OR trans-scrotal approach). Both the Mesh Term search and keyword search were combined. In addition, the reference lists were manually retrieved to broaden the search.

### Study Selection and Data Extraction

The inclusion criteria for our systematic review and meta-analysis were (1) clinical trials comparing the SISO and TTIO surgical strategy regardless of prospective or retrospective designed studies and (2) children who were diagnosed with primary PUDTs and (3) have a follow-up time of more than 3 months and adequate initial data available. Exclusion criteria were (1) no TTIO as a control group, (2) secondary cryptorchidism or has a history of former orchiopexy or inguinal operation, and (3) studies that did not provide sufficient data or repeated or duplicate publications. Only the latest study was included if there were more than one report paper from one clinical center or duplicate publications detected.

All included initial trials were thoroughly understood and analyzed; the baseline characteristics summarized were authors, publication years, countries, study periods and designs, age of participants, single-incision scrotal techniques (high transverse stria scrotal orchiopexy or low trans-scrotal mid raphe orchiopexy), laterality, and location of testes. Operative time, hospitalization duration, patent processus vaginalis, short-term complications (wound infection or dehiscence, scrotal hematoma or severe swelling), and long-term complications (testicular atrophy, testicular reascent, hernia, or hydrocele) were extracted to compare these two surgical approaches for PUDT. We also made possible comparisons between each single-incision technique and conventional two-incision orchiopexy. Moreover, conversion rates, postoperative analgesics needs, and cosmetic results were also concerned. Cometic results were only evaluated upon exerted operation scars. Two reviewers (CJY and YH) independently conducted the literature search, screening, and data extraction. All discrepancies were resolved by discussion or, still unresolved, by a mediating reviewer (SDW).

### Quality Assessment

There was no best suitable tool to assess the quality of included studies in this study. For compromising reasons, the methodological quality of retrospective case–control studies was assessed using the Newcastle–Ottawa Quality Assessment Scale (NOS), and the prospective randomized controlled trials were assessed using the Jadad Scale. This NOS used a star system (measured 0–9) to assess the quality of a study in three domains: the selection of the study groups, the comparability of the groups, and the ascertainment of exposure of interest for case–control studies. Also, the Jadad Scale evaluated the quality of a randomized study by randomization, blinding, and withdrawals, which marked 0–5; 1–2 indicated low quality and 3–5 high quality.

### Statistical Analysis

All statistical data analyses were completed by Review Manager version 5.3 (Cochrane Collaboration, Oxford, United Kingdom). The odds ratios (ORs) and 95% confidence intervals (CI) were calculated for dichotomous variables using the Mantel–Haenszel method. In addition, for continuous variables, mean difference (MD) and 95% CI were applied using the inverse-variance method. The pooled heterogeneity of included studies was tested using both the chi-square test (*p* ≥ 0.1, indicating low heterogeneity) and I^2^ index statistics (0%, indicating no inter-study heterogeneity) (18). The fixed-effect model was applied if I^2^ <50%; otherwise, the random effect model was used (19). Sensitivity analysis was conducted by removing a study per circle to detect the heterogeneity contribution of each study. Funnel plots visually detected publication basis. A *P* < 0.05 was considered statistically significant in this study.

## Results

### Identification and Eligibility of Studies

**Figure 1:**
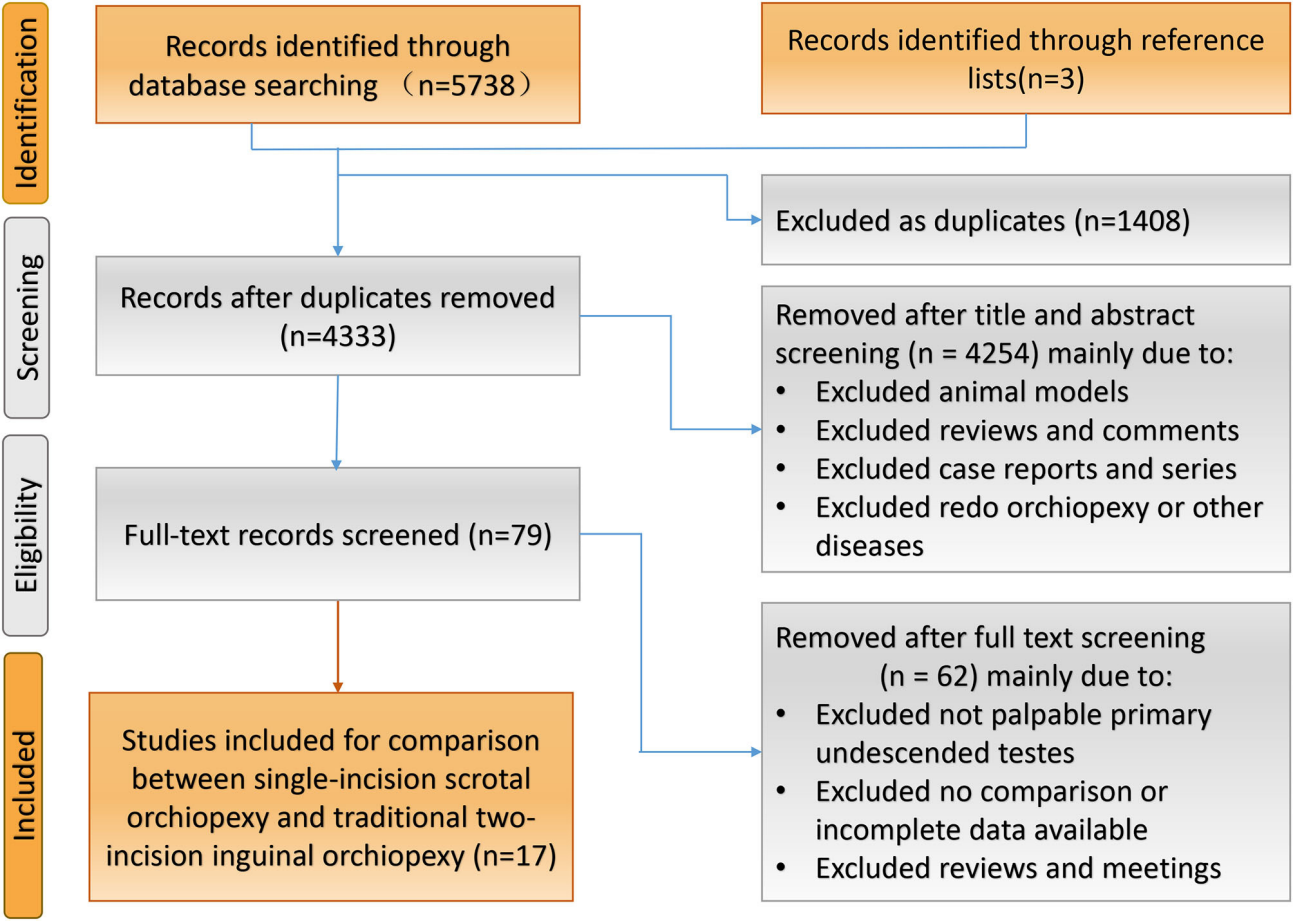
Flow diagram of identification and eligibility of initial publications.

Initial electronic databases search yielded 5,738 possible articles (Figure 1). In addition, three papers were added from a manual search of the retrieved articles and relevant reviews. A total of 4,333 titles and abstracts were screened after eliminating 1,408 duplicates. Eventually, only 17 studies involving 2,627 children (1,362 underwent SISOs and 1,265 TTIOs) met our inclusion criteria after a comprehensive full-text screen. There were seven prospective trials (6, 12, 15, 20–23) and 10 retrospective studies (10, 11, 13, 14, 24–29).

The baseline characteristics of included studies are summarized in Table 1. Tables 2, 3 demonstrate the extracted data information of the operative time, hospitalization duration, patent processus vaginalis, short-term complications (wound infection or dehiscence, scrotal hematoma or severe swelling), long-term complications (testicular atrophy, testicular reascent, hernia, or hydrocele), postoperative analgesics needs, and cosmetic result between SISO and TTIO for PUDT in detail.

**Table 1:** Baseline characteristics of these included 17 initial studies comparing single-incision scrotal orchiopexy and traditional two-incision inguinal orchiopexy for primary palpable undescended testes.

**Authors**	**Country/** **zone**	**Study periods**	**Study design**	**Scrotal operation,** **transverse stria or** **mid raphe^$$^**	**Type of** **cryptorchidism**	**Age, Mean ±SD** **(range), year**		**Laterality (right/left or** **unilateral/bilateral)**		**Location (external ring or** **below/inguinal canal)**	
						**SISO**	**TTIO**	**SISO**	**TTIO**	**SISO**	**TTIO**
Al-Mandil et al. (10)	Canada	January 2004 to March 2007	Age matched case control study	High transverse stria scrotal orchiopexy	Primary unilateral palpable UDT	4.6	4.7	30/33	29/24	26/37	21/32
Badbarin et al. (20)	Iran	March 2014 to March 2015	Randomized controlled study	Single incision trans-scrotal orchiopexy, NS	Palpable Low-Lying Undescended Testis	3.58 ± 2.66 (1–14)	2.57 ± 2.03 (5–12)	29/1	26/4	NR	NR
Ben Dhaou et al. (21)	Tunisie	January 2011 to December 2013	Prospective study	High transverse stria scrotal orchidopexy	Palpable undescended testis	3.82 ± 0.75	4.49 ± 1.08	78/11	60/20	NR	NR
Chen et al. (24)	China	January 2015 to December 2015	Retrospective study	Low trans-scrotal mid raphe orchiopexy	Palpable undescended testis	1.5 (0.6–7.0)	1.4 (0.8–11)	44/9	49/7	17/45	12/51
Cloutier et al. (11)	Canada	January 2003 to September 2009	Retrospective cohort study	Low trans-scrotal mid raphe orchiopexy, high scrotal incision	Low palpable undescended testis	Low 5.25 ± 2, high 4.42 ± 1.92	2.17 ± 0.92	Low 37/44, high 28/16	77/12	NR	NR
Cuda et al. (25)	USA	January 2002 to July 2009	Retrospective study	Single incision scrotal orchiopexy, NS	Palpable undescended testis	NR	NR	233/16	162/8	NR	NR
Duan et al. (29)	China	July 2011 to July 2013	Retrospective study	Low trans-scrotal mid raphe orchiopexy	Palpable undescended testes	NR	NR	NR	NR	NR	NR
Eltayeb (12)	Egypt	November 2009 to October 2013	Randomized controlled study	High transverse stria scrotal incision	Unilateral palpable undescended testes	0.83–6	0.83–6	NR	NR	11/24	13/22
Lee et al. (13)	Korea	April 2004 to April 2008	Retrospective study	Single scrotal incision, NS	Palpable undescended teste	3.12 ± 1.9	2.56 ± 1.92	NR	NR	21/4	28/4
McGrath et al. (15)	Canada	January-2015 to June 2019	Randomized controlled study	High transverse stria scrotal incision	Unilateral palpable undescended testis	2.18 ± 1.37	2.27 ± 1.47	38/42	52/29	30/50	23/58
Na et al. (22)	Korea	January 2007 to December 2010	Prospective randomized controlled study	Transverse stria scrotal incision orchiopexy	Palpable low-lying undescended testis	3.34 ± 0.86	3.48 ± 0.95	68/39	69/36	86/21	82/23
Nazem et al. (6)	Iran	May 2017 to May 2018	Randomized single-blind study	Trans-scrotal single incision orchiopexy, NS	Palpable undescended testis	0.85 ± 0.17	0.85 ± 0.14	36/14	44/6	NR	NR
Ramzan et al. (23)	Pakistan	April 2007 to April 2010	Randomized controlled study	Low transverse stria scrotal incision orchiopexy	Palpable undescended testes	NR	NR	NR	NR	NR	NR
Sutton et al. (26)	UK	1998 to 2008	Retrospective study	Single scrotal incision orchidopexy, NS	Palpable undescended testes	5.5 (4.7–6.3)	NR	NR	NR	NR	NR
Takahashi et al. (14)	Japan	July 1998 to June 2005	Retrospective study	Low transverse stria scrotal incision orchiopexy	Undescended testis located distal to the external inguinal ring	NR	NR	NR	NR	NR	NR
Wang et al. (27)	China	October 2008 to December 2009	Retrospective study	High transverse stria scrotal incision orchiopexy	Palpable undescended testes	2.5 (1.5–6)	2.5 (0.5–6)	24/4	25/5	18/14	19/16
Yi et al. (28)	China	March 2006 to May 2011	Retrospective study	Low trans-scrotal mid raphe orchiopexy	Palpable undescended testes	5.4	5.5	33/3	33/3	NR	NR

*SISO indicates single-incision scrotal orchiopexy; TTIO, traditional two-incision inguinal orchiopexy; NR, not reported; NS, not specified*.

*^$$^ These two single-incision orchiopexy techniques: high transverse stria scrotal incision and low trans-scrotal mid raphe orchiopexy*.

**Table 2:** Sample size, operative time, hospitalization duration, patent processus vaginalis, total complications and conversions between single-incision scrotal orchiopexy and traditional two-incision inguinal orchiopexy.

	**Sample size,** **patients/testes**		**Operative time,** **Mean ±SD, min**		**Hospitalization duration,** **day**		**Patent processus** **vaginalis**		**Total complication**		**Conversion** **to TTIO**
	**SISO**	**TTIO**	**SISO**	**TTIO**	**SISO**	**TTIO**	**SISO**	**TTIO**	**SISO**	**TTIO**	
Al-Mandil et al. (10)	63	53	34	64	NA	NA	NA	NA	4	2	0
Badbarin et al. (20)	30	30	19.06 ± 2.96	30 ± 10.42	NA	NA	NA	NA	7	7	0
Ben Dhaou et al. (21)	89/100	80/100	21.7 ± 10.2	32.03 ± 9	NA	NA	NA	NA	12	12	0
Chen et al. (24)	53/62	56/63	32 ± 8.75	46 ± 15	NA	NA	NA	NA	2	1	3
Cloutier et al. (11)	125/185^&&^	89/101	28 ± 10	37 ± 12	NA	NA	37	69	3	2	1
Cuda et al. (25)	249/265	170/178	NA	NA	NA	NA	NA	NA	5	10	0
Duan et al. (29)	40	40	23.28 ± 7.69	35.48 ± 8.21	3.01 ± 1.18	5.43 ± 1.26	NA	NA	0	4	0
Eltayeb (12)	35	35	18.12 ± 4.21	25.58 ± 6.47	NA	NA	23	23	5	1	3
Lee et al. (13)	25	32	39.76 ± 7.6	53.31 ± 6.3	NA	NA	21	26	3	5	1
McGrath et al. (15)	80	81	30.6 ± 12	34.5 ± 9.5	2.2 ± 0.58	2.3 ± 0.78	NA	NA	5	1	19
Na et al. (22)	107	105	40.5 ± 25.9	62.3 ± 35.6	2.1 ± 0.8	2.5 ± 0.7	NA	NA	2	1	9
Nazem et al. (6)	50/64	50/56	30.24 ±19.16	70.74 ± 7.42	2.03 ± 0.88	2.41 ± 0.76	12	6	9	10	0
Ramzan et al. (23)	134	135	28.32 ± 0.92	47.83 ± 0.76	1.03 ± 0.21	3.02 ± 0.20	NA	NA	8	14	10
Sutton et al. (26)	55	75	29.5 ± 18.1	42.7 ± 16.6	NA	NA	NA	NA	2	4	3
Takahashi et al. (14)	46	107	45.2 ± 10.75	66.6 ± 30.5	NA	NA	14	NR	1	0	0
Wang et al. (27)	28/32	30/35	20 ± 7	35 ± 6	3	5	NA	NA	5	2	0
Yi et al. (28)	36/39	36/39	33	41	NA	NA	NA	NA	0	0	0

*SISO indicates single-incision scrotal orchiopexy; TTIO, traditional two-incision inguinal orchiopexy; NA, not available*.

*^&&^81/125 low scrotal single-incision orchiopexy and 44/60 high scrotal single-incision orchiopexy*.

**Table 3:** The short-term and long-term exact complications, postoperative pain and cosmetic results in these two approaches for primary palpable undescended testes.

	**Wound infection or** **dehiscence**		**Hematoma or severe** **swelling**		**Testicular atrophy**		**Testicular re-ascent**		**Hernia or hydrocele**		**Postoperative** **pain**	**Scar and** **cosmetic results**
	**SISO**	**TTIO**	**SISO**	**TTIO**	**SISO**	**TTIO**	**SISO**	**TTIO**	**SISO**	**TTIO**		
Al-Mandil et al. (10)	1	1			0	0	1	1	2	0	SISO less pain, though not objectively measured	
Badbarin et al. (20)	1	0	6	0	0	2	0	5				100% satisfactory for SISO,76% satisfactory for TTIO
Ben Dhaou et al. (21)	0	1	4	8	0	0	8	3				
Chen et al. (24)	0	0	2	0	0	0	0	1	0	0		
Cloutier et al. (11)	0	2	1	0	0	0	2	0				
Cuda et al. (25)					0	4	4	5	1	1		
Duan et al. (25)	0	3					0	1				
Eltayeb (12)			2	0	1	0	2	1	0	0	No postoperative potent-analgesics were needed	SISO better, no scar scale done
Lee et al. (13)	2	4			0	0	1	1	0	0		SISO has more cosmetically appealing results
McGrath et al. (15)	1	1			1	0	3	0			SISO less pain, quantificationally evaluated	
Na et al. (22)	1	1	1	0	0	0	0	0				Satisfaction with the cosmetic result was 96.6%and 96.5%
Nazem et al. (6)	2	1			5	4	1	3	1	2		
Ramzan et al. (23)												
Sutton et al. (26)	1	3	1	0	0	0	0	1	0	0		
Takahashi et al. (14)	0	0	0	0	0	0	1	0	0	0		
Wang et al. (27)	0	0			0	0	3	1	2	1		SISO more cosmetically appealing results
Yi et al. (28)	0	0	0	0	0	0	0	0	0	0		

*SISO indicates single-incision scrotal orchiopexy; TTIO, traditional two-incision inguinal orchiopexy*.

### Quality Assessment

The quantitative star NOS scores for retrospective case–control studies ranged from 4 to 6, indicating low to moderate quality (Supplementary Table 1). The Jadad score scale for seven randomized controlled studies ranged from 2 to 5, whereas only two of seven was assessed as high quality (with a score of 3 or 5). Among these seven studies, withdrawals were well-adopted, and the randomization and double-blind method were still a big challenge. For some way, the double-blind method would be greatly restricted by the informed consent of participants and surgical strategy-designed trials.

### Operative Time, Hospitalization Duration, and Patent Processus Vaginalis

**Figure 2:**
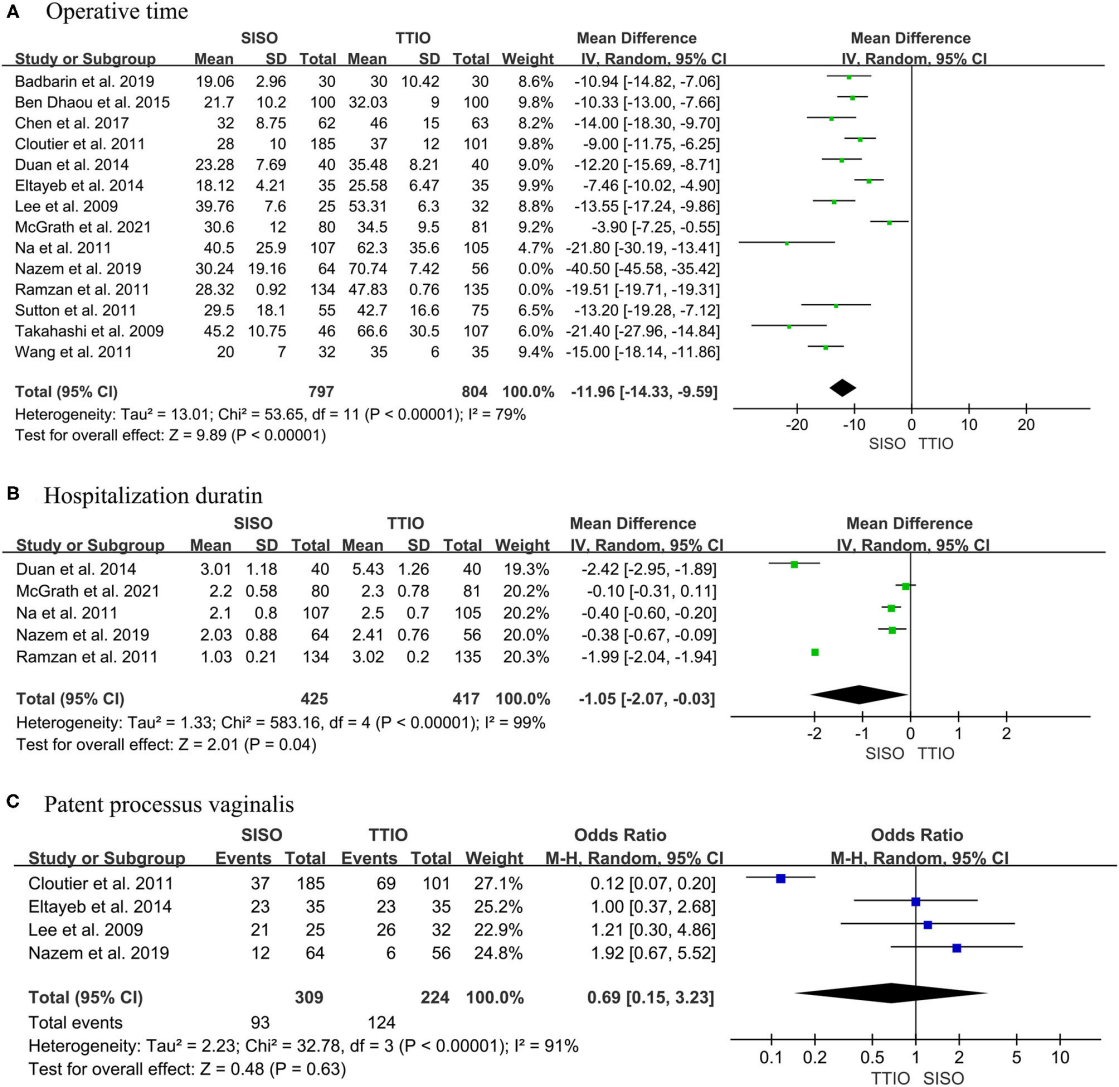
Forest plot of **(A)** operative time, **(B)** hospitalization duration, and **(C)** patent processus vaginalis between single-incision scrotal orchiopexy and traditional two-incision inguinal orchiopexy for primary palpable undescended testes.

Fourteen initial studies compared the operative time between SISO and TTIO for PUDT (6, 11–15, 20–24, 26, 27, 29). High heterogeneity (I^2^ = 97%) was observed, so the random effect model was used (19). Sensitivity analysis revealed that two studies contributed too much heterogeneity; these two studies were removed in the meta-analysis, although this removal would not affect the main results (6, 23). Our meta-analysis yielded that the SISO approach had a statistically significant shorter operative time than the TTIO approach for PUDT (WMD−11.96, 95% CI−14.33 to−9.59, I^2^ = 79%, *P* < 0.00001) (Figure 2A).

Five studies reported hospitalization duration (6, 15, 22, 23, 29). The same high heterogeneity was observed, so the random effect was applied (I^2^ = 99%). Meta-analysis revealed that the SISO had a significantly shorter postoperative hospital duration compared with TTIO (WMD−1.05, 95% CI−2.07 to −0.03, *P* = 0.04) (Figure 2B).

The incidence of patent processus vaginalis was reported in four studies (6, 11–13). Our finding demonstrated no significant differences between these two surgical approaches, even with high heterogeneity between studies (OR 0.69, 95% CI 0.15 to 3.23, I^2^ = 91%, *P* = 0.63) (Figure 2C).

### Complications

**Figure 3:**
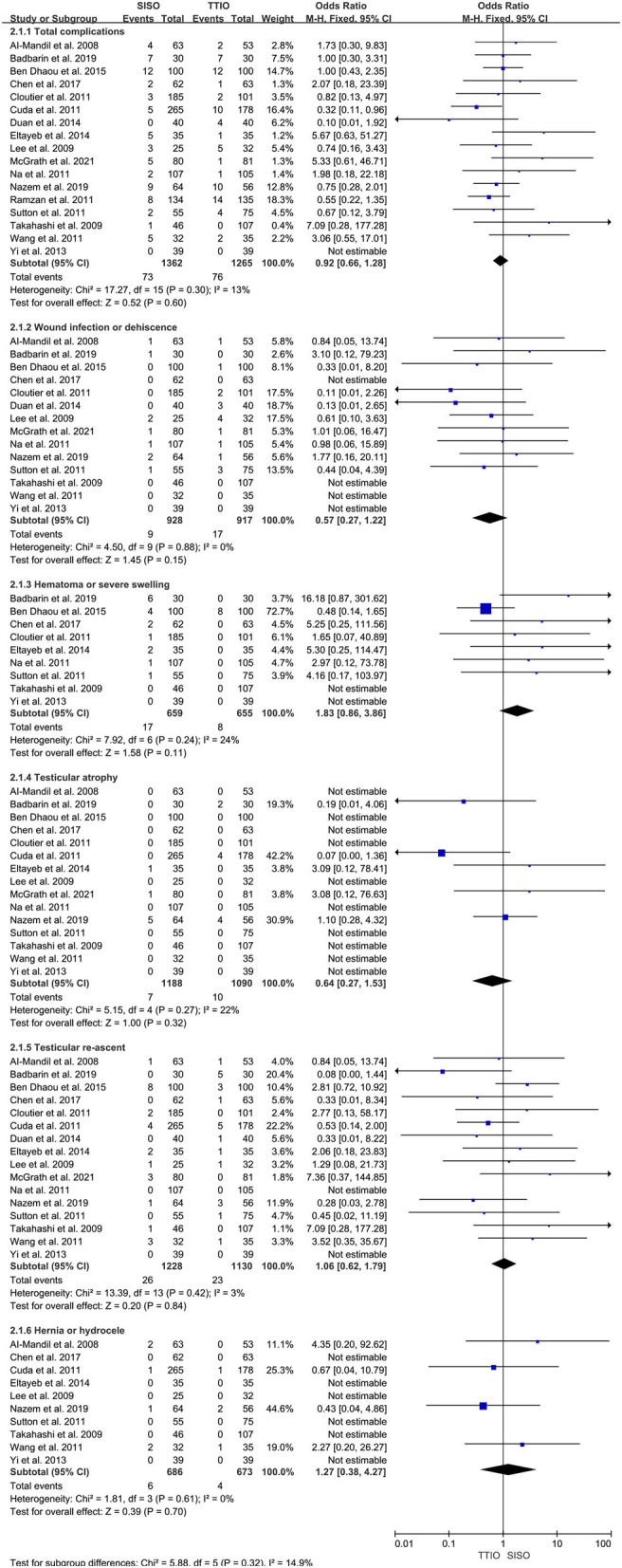
Forest plot of total, short-term, and long-term complications between single-incision scrotal orchiopexy and traditional two-incision inguinal orchiopexy for primary palpable undescended testes.

All of these include studies reported about complications. Generally, there were 73 (5.4%) total complications in SISO and 76 (6.0%) in the TTIO group. Although the results were comparable, our analysis demonstrated no significant differences between SISO and TTIO (OR 0.92, 95% CI 0.66 to 1.28, I^2^ = 13%, *P* = 0.60) (Table 3, Figure 3).

Wound infection/dehiscence and scrotal hematoma/severe swelling were regarded as short-term complications. There were nine cases (1.0%) who suffered from wound infection or dehiscence in SISO and 17 cases (1.9%) in the TTIO group, whereas meta-analysis demonstrated no statistically significant difference (OR 0.57, 95% CI 0.27 to 1.22, I^2^ = 0%, *P* = 0.15). There were 17 cases (2.6%) who suffered from scrotal hematoma or severe swelling in SISO and 8 cases (1.2%) in the TTIO group; meta-analysis demonstrated no significant difference (OR 1.83, 95% CI 0.86 to 3.86, I^2^ = 24%, *P* = 0.11) (Figure 3).

Testicular atrophy, reascent, hydrocele, or hernia were regarded as long-term complications. There were 15, 16, and 10 studies that reported testicular atrophy, reascent, and hydrocele or hernia, respectively. In total, there were 7 cases (0.6%) who developed into testicular atrophy in SISO and 10 (0.9%) in the TTIO group; meta-analysis revealed no significant difference (OR 0.64, 95% CI 0.27 to 1.53, I^2^ = 22%, *P* = 0.32). Twenty-six cases (2.1%) and 23 cases (2.0%) were detected of testicular reascent in SISO and TTIO, respectively, the same, without statistically significant difference (OR 1.06, 95% CI 0.62 to 1.79, I^2^ = 3%, *P* = 0.84). Also, a total of 6 cases (0.9%) and 4 cases (0.6%) were detected of hernia or hydrocele during follow-up in SISO and TTIO, respectively, without significant difference (OR 1.27, 95% CI 0.38 to 4.27, I^2^ = 0%, *P* = 0.70) (Figure 3).

There was no evidence for high heterogeneity for complications was observed, be it short-term, long-term, or total, so the fixed effect model was applied.

### Postoperative Pain Evaluation, Cosmetic Results, and Conversions

All papers reported the conversion status of SISO to TTIO. Added in total, 49 cases (3.6%) needed an extra inguinal incision to assist dissection of the spermatic cord or high ligation of processus vaginalis, and the majority of these testes were intracanalicular. What is more, nine studies (52.9%) did not report the need for any conversion in their surgical practice (Table 2).

Three studies evaluated postoperative pain, whereas only one study quantitatively calculated analgesics intake and pain scale assessment (15). It could be concluded that both SISO and TTIO did not need many analgesics after an operation, and relatively speaking, SISO consumed less. There were five studies that evaluated scar and cosmetic appealing satisfactory of patients. All these studies had the same conclusion that SISO provided more cosmetically appealing results than TTIO (Table 3).

## Discussion

Our updated systematic review and meta-analysis combined the most evidence comparing SISO and TTIO for primary PUDT. We analyzed the differences in aspects of operative time, hospitalization duration, conversion rates, wound infection or dehiscence, scrotal hematoma or severe scrotal, testicular atrophy, reascent, hernia or hydrocele, postoperative analgesics needs, and cosmetic results between these two surgical approaches for PUDT. According to our meta-analysis, SISO had the advantages of shorter operative time and hospital stay, fewer analgesics needs, and better cosmetic appealing results, most importantly, in the meanwhile, did not increase the short-term and long-term complications rates. The total conversion rate of SISO was 3.6%, and the majority of which were intracanalicular. Taking all these evidence and practice into consideration, we recommend that SISO should be the first choice for primary PUDT, especially low palpable cryptorchidism.

Currently, SISO mainly contains a single high transverse stria scrotal incision and low trans-scrotal mid raphe orchiopexy (11, 30). There was also low transverse stria scrotal incision orchiopexy introduced (14). Both high transverse stria scrotal incision and low trans-scrotal mid raphe had shorter operation time than TTIO, and mid raphe incision showed shorter time than stria scrotal incision (*P* < 0.00001, Supplementary Figure 1). Transverse stria scrotal incision showed more total complications than TTIO (*P* = 0.03, Supplementary Figure 2); however, differences between mid raphe orchiopexy and TTIO did not reach significance. Intuitively, both surgical technologies have less incision to close, and the incisions are invisible, and this makes SISO have shorter operative time and better cosmetic results than TTIO, although this only concerns the exerted operation scars. In addition, SISO does not open the external oblique fascia and has less dissection and anatomical disruption of the inguinal region, which would undoubtedly account for less postoperative pain and shorter hospitalization duration (10).

The main worrisome trouble of SISO for undescended testes is the difficulty for dissection of the spermatic cord and high ligation of processus vaginalis or hernia sac. This is also why TTIO lasts so long while still classic in the history of orchiopexy for cryptorchidism and could be served as the rescue method for SISO. In contrast, in a recent study, Hyuga et al. concluded that ligation of the processus vaginalis is unnecessary when it is not widely patent (31). In our opinion, this conclusion still needs to be interpreted with great caution, and studies on this topic still have a long way to go.

SISO for treatment of primary, secondary, or even trapped testes can be well-tolerated (4). Furthermore, it was reported that a single-incision scrotal surgical procedure had been successfully applied to treat communicating hydroceles and indirect hernia, or even impalpable cryptorchidism (10, 32). This procedure has its own advantages superiority, and we do believe it would serve the urologists better in more aspects in the future. However, urologists and every surgeon must take their own learning experiences and familiarity with surgical strategy into the deep heart.

There are several surgical techniques to correct cryptorchidism. Cuda et al. reported their clinical experience that the rate of laparoscopic and scrotal orchiopexy increased, whereas the inguinal orchiopexy decreased (25). The laparoscopic technique gradually tends to have fewer incisions and even a single port, and the single practice trend is becoming more and more warrant in minimal invasive surgery (33). SISO meets the concept of being minimally invasive and the demands of patients; it is a safe, effective, promising, and potential surgical approach alternative to TTIO.

Our study still has some limitations that must be taken into consideration. Most importantly, high heterogeneity between studies should not be ignored. Secondly, the small sample size, low quality of the initial study, and different study designs would make the analysis somehow weaker. Third, it was impossible to match all participated children groups with age, body mass index, the accurate location of the testis, and anatomic variations. Fourth, publication bias was still a big limitation, although the funnel plot was evaluated to be acceptable. In addition, selection bias for choosing the techniques cannot really be addressed in these studies, i.e., inguinal orchiopexy for higher located testicles and single incision for the lower ones. For example, 52.9% (9/17) studies reported there was no need for conversion, whereas a randomized controlled study reported that the conversion rate of SISO was as high as 23.8% (15). Alyami et al. also pointed out that only 52.8% of surgeons used SISO for undescended testes, and there was a discrepancy in the reported advantages and success rate according to their investigation (34). Last but not least, testicle positions were not well-evaluated, and only in the low-inguinal undescended testis can the cremaster dissection be adequate for excellent results. On the contrary, the correction of the high-inguinal undescended testis may need high ligation of the processus vaginalis or hernia sac and more extended dissection of the spermatic cord until the level of the internal inguinal ring, and it can provoke more tissue damage compared with the opening of the external oblique fascia. Furthermore, we cannot randomly ignore the high incidence of ipsilateral and contralateral patent processus vaginalis or hernia, especially in lower positioned cryptorchid patients (35). Added in total, SISO should only be applied in highly selected cases. More well-designed, high-quality, large-scale, multicenter prospective trials are still needed to explain this.

## Conclusion

SISO is a safe, effective, promising, and potential minimal invasive surgical approach for PUDT. Compared with TTIO, SISO has the advantages of shorter operative time, shorter hospitalization duration, less postoperative pain, better cosmetic appealing results, and not increasing short-term and long-term complications. SISO is an alternative to TTIO in selected cryptorchid patients, especially lower positioned ones.

## Data Availability Statement

The original contributions presented in the study are included in the article/Supplementary Material, further inquiries can be directed to the corresponding author/s.

## Author Contributions

CY and SW conceived and designed the meta-analysis. CY and LK independently searched the electronic databases and independently extracted the data. CY led analysis and interpretation of data, drafted the manuscript, and revised content based on feedback. LK acted as the second reviewer. YH and LW assisted with the retrieval of database and acquisition of data. JZ and JL assisted with the interpretation of data and provided critical revision of drafts. TL, DH, and GW assisted with conception and design and provided critical revision of drafts. SW acted the role of the corresponding author, provided funding support, provided critical revision of drafts, and acted as the third (mediating) reviewer. All authors contributed to the article and approved the submitted version.

## Funding

This study was supported by the National Natural Science Foundation of China (No. 81873828), the Scientific and Technological Research Program of Chongqing Municipal Education Commission (KJ1600229), the Postgraduate Scientific Research Innovation Project of Chongqing Medical University (CYB16105), and the Senior Medical Talents of Chongqing for Young and Middle-Aged.

## Conflict of Interest

The authors declare that the research was conducted in the absence of any commercial or financial relationships that could be construed as a potential conflict of interest.

## Publisher's Note

All claims expressed in this article are solely those of the authors and do not necessarily represent those of their affiliated organizations, or those of the publisher, the editors and the reviewers. Any product that may be evaluated in this article, or claim that may be made by its manufacturer, is not guaranteed or endorsed by the publisher.

## Supplementary Material

Supplementary Figure 1 The forest plot of comparison of operation time between high transverse stria single-incision scrotal orchiopexy and low trans-scrotal mid raphe single-incision orchiopexy and traditional two-incision inguinal orchiopexy.

Supplementary Figure 2 The forest plot of comparison of total complications between high transverse stria single-incision scrotal orchiopexy and low trans-scrotal mid raphe single-incision orchiopexy and traditional two-incision inguinal orchiopexy.

Supplementary Table 1 Quality assessment of included retrospective designed case control studies according to Newcastle–Ottawa Quality Assessment Scale (NOS), and randomized controlled trials according to Jadad Scale.

## Abbreviations

CI: confidence interval
OR: odds ratio
PUDTs: palpable undescended testes
SISO: single-incision scrotal orchiopexy
TTIO: traditional two-incision inguinal orchiopexy.

## References

[1] Braga LH Lorenzo AJ Romao RLP. Canadian urological association-pediatric urologists of Canada (CUA-PUC) guideline for the diagnosis, management, and followup of cryptorchidism. Can Urol Assoc J. (2017) 11:E251. 10.5489/cuaj.4585 28761584 PMC5519382

[2] Levine H Keinan-Boker L Leiba A Derazne E Rais A Kark JD. Paternal age and risk of testicular germ cell tumors: a cohort study of 1,000,000 men. Andrology. (2017) 5:1124. 10.1111/andr.12422 28950439

[3] Wang C Wang Y Chen X Wei X Chen F Zhong M. Efficacy of single-stage and two-stage Fowler-Stephens laparoscopic orchidopexy in the treatment of intraabdominal high testis. Asian J Surg. (2017) 40:490. 10.1016/j.asjsur.2016.11.008 28410943

[4] Caruso A Walsh R Wolach J Koyle MA. Single scrotal incision orchiopexy for the palpable undescended testicle. J Urol. (2000) 164:156. 10.1016/S0022-5347(05)67485-X 10840452

[5] Ritchey M Bloom D. Modified dartos pouch orchiopexy. Urology. (1995) 45:136. 10.1016/S0090-4295(95)97502-0 7817467

[6] Nazem M Hosseinpour M Alghazali A. Trans-scrotal incision approach versus traditional trans-scrotal incision orchiopexy in children with cryptorchidism: a randomized trial study. Adv Biomed Res. (2019) 8:34. 10.4103/abr.abr_26_19 31259163 PMC6543866

[7] Karaman I Karaman A Erdogan D Çavuşoglu Y Cavuşglu Y. The transscrotal approach for recurrent and iatrogenic undescended testes. Eur J Pediatr Surg. (2010) 20:267. 10.1055/s-0030-1249048 20225179

[8] Dayanç M Kibar Y Tahmaz L Yildirim I Peker A. Scrotal incision orchiopexy for undescended testis. Urology. (2004) 64:1216. 10.1016/j.urology.2004.06.069 15596200

[9] Lin J Li D Chen J Lin L Xu Y. Inguinal hernia repair by Bianchi incision in boys: a retrospective study. Pediatr Surg Int. (2018) 34:289. 10.1007/s00383-017-4217-x 29188379

[10] Al-Mandil M Khoury AE Ei-Hout Y Kogon M Dave S Farhat W. Potential complications with the prescrotal approach for the palpable undescended testis? a comparison of single prescrotal incision to the traditional inguinal approach. Urol J. (2008) 180:686. 10.1016/j.juro.2008.04.040 18554646

[11] Cloutier J Moore K Nadeau G Bolduc S. Modified scrotal (Bianchi) mid raphe single incision orchiopexy for low palpable undescended testis: early outcomes. J Urol. (2011) 185:1088. 10.1016/j.juro.2010.10.039 21255806

[12] Eltayeb AA. Single high scrotal incision orchidopexy for unilateral palpable testis: a randomised controlled study. Afr J Paediatr Surg. (2014) 11:143. 10.4103/0189-6725.132808 24841015

[13] Lee Hr Lee YS Kim HS Lee JY Kim JC Koh JS. A Comparison between single scrotal incision orchiopexy and the inguinal approach in patients with palpable undescended testes distal to the external inguinal ring. Korean J Urol. (2009) 50:1133. 10.4111/kju.2009.50.11.1133

[14] Takahashi M Kurokawa Y Nakanishi R Koizumi T Yamaguchi K Taue R . Low transscrotal orchidopexy is a safe and effective approach for undescended testes distal to the external inguinal ring. Urol Int. (2009) 82:92. 10.1159/000176033 19172105

[15] McGrath M Kim J Farrokhyar F Braga L. Randomized controlled trial of scrotal versus inguinal orchidopexy on postoperative pain. Urol J. (2021) 205:895. 10.1097/JU.0000000000001379 33021443

[16] Bassel Y Scherz H Kirsch A. Scrotal incision orchiopexy for undescended testes with or without a patent processus vaginalis. Urol J. (2007) 177:1516. 10.1016/j.juro.2006.11.075 17382769

[17] Feng S Yang H Li X Yang J Zhang J Wang A . Single scrotal incision orchiopexy versus the inguinal approach in children with palpable undescended testis: a systematic review and meta-analysis. Pediatr Surg Int. (2016) 32:989. 10.1007/s00383-016-3956-4 27510940

[18] Gurney J Richiardi L McGlynn K Signal V Sarfati D. Analgesia use during pregnancy and risk of cryptorchidism: a systematic review and meta-analysis. Hum Reprod. (2017) 32:1118. 10.1093/humrep/dex047 28333256 PMC5808643

[19] Higgins JP Thompson SG Deeks JJ Altman DG. Measuring inconsistency in meta-analyses. BMJ. (2003) 327:557. 10.1136/bmj.327.7414.557 12958120 PMC192859

[20] Badbarin D Mousavi Toomatari SE Aslanabadi S Farhadi E Akhavan Salamat S. A comparative study of single scrotal incision orchiopexy of children with palpable low-lying undescended testis with traditional inguinal method. Adv Pediatr Surg. (2019) 25:14. 10.13029/aps.2019.25.1.14

[21] Ben Dhaou M Zouari M Zitouni H Jallouli M Mhiri R. [Comparison of the inguinal and scrotal approaches for the treatment of cryptorchidism in children]. Prog Urol. (2015) 25:598. 10.1016/j.purol.2015.05.005 26094098

[22] Na SW Kim SO Hwang EC Oh KJ Jeong SI Kang W . Single scrotal incision orchiopexy for children with palpable low-lying undescended testis: early outcome of a prospective randomized controlled study. Korean J Urol. (2011) 52:637. 10.4111/kju.2011.52.9.637 22025961 PMC3198239

[23] Ramzan M Sheikh AH Qureshi MA Zubair M Majid F. Single incision trans scrotal versus standard inguino-scrotal orchidopexy in children with palpable undescended testis: our experience from April 2007 to April 2010. Pak J Med Sci. (2012) 28:827.

[24] Chen Y Zhao JF Wang FR Li Y Shi Z Zhong HJ . Single scrotal-incision orchidopexy without ligation of processus vaginalis for palpable undescended testis. Zhonghua Nan Ke Xue. (2017) 23:708–12. 29726645

[25] Cuda SP Srinivasan AK Kalisvaart J Kirsch AJ. Evolution of single practice trends in the surgical approach to the undescended testicle. J Urol. (2011) 185:2451. 10.1016/j.juro.2011.01.010 21555009

[26] Sutton PA Greene OJ Adamson L Singh SJ. Scrotal fixation in the management of low undescended testes. J Indian Assoc Pediatr Surg. (2011) 16:142. 10.4103/0971-9261.86871 22121312 PMC3221156

[27] Wang JJ Tang DX Wu DX Tao C Yang HJ. Single scrotal incision orchiopexy (Bianchi technique) for medium and low undescended testicle [In Chinese]. Chin J Pediatr Surg. (2011) 32:354.

[28] Yi QJ He DW Bian ZD Li XL Liu JH Liu F . Comparison of single prescrotal incision and traditional inguinal orchiopexy [In Chinese]. Chin J Urol. (2013) 34:120.

[29] Duan ZN Lu ZH Mo SB. Clinical effect observation of tran-scrotal dermatoglyph signle incision in the treatment of palpable cryptorchidism in children. China Mod Med. (2014) 21:180.

[30] Ali MS Khan N Uddin MB Hossain MS Mushtabshirah L. High transverse scrotal incision orchiopexy for undescended testes. Mymensingh Med J. (2019) 28:542–46. 31391424

[31] Hyuga T Kawai S Nakamura S Kubo T Nakai H. Long-term outcome of low scrotal approach orchiopexy without ligation of the processus vaginalis. J Urol. (2016) 196:542. 10.1016/j.juro.2016.02.2962 26944301

[32] Callewaert PR Rahnama'i MS Biallosterski BT van Kerrebroeck PE. Scrotal approach to both palpable and impalpable undescended testes: should it become our first choice? Urology. (2010) 76:73. 10.1016/j.urology.2009.09.096 20156655

[33] Ma Y Cai J Li S Wang W Liu L. Single-port laparoscopic assisted transcrotal orchidopexy for palpable inguinal canalicular cryptorchidism accompany with indirect inguinal hernia. Front Pediatr. (2018) 6:293. 10.3389/fped.2018.00293 30356669 PMC6189310

[34] Alyami FA Bin Yahya AF Albraidi HF Almarek NA Alkhalifa MA Alhazmi H . Utilization of scrotal orchidopexy for palpable undescended testes among surgeons. Urol Ann. (2018) 10:380. 10.4103/UA.UA_54_18 30386090 PMC6194784

[35] Aggarwal H Kogan BA Feustel PJ. One third of patients with a unilateral palpable undescended testis have a contralateral patent processus. J Pediatr Surg. (2012) 47:1711. 10.1016/j.jpedsurg.2012.01.003 22974611

