# Supplementary figures and images for: Comparison of Single-Incision Scrotal Orchiopexy and Traditional Two-Incision Inguinal Orchiopexy for Primary Palpable Undescended Testis in Children: A Systematic Review and Meta-Analysis
Source: Front Pediatr. 2022 Mar 15;10:805579. doi: 10.3389/fped.2022.805579 (PMC8964791; doi:10.3389/fped.2022.805579)

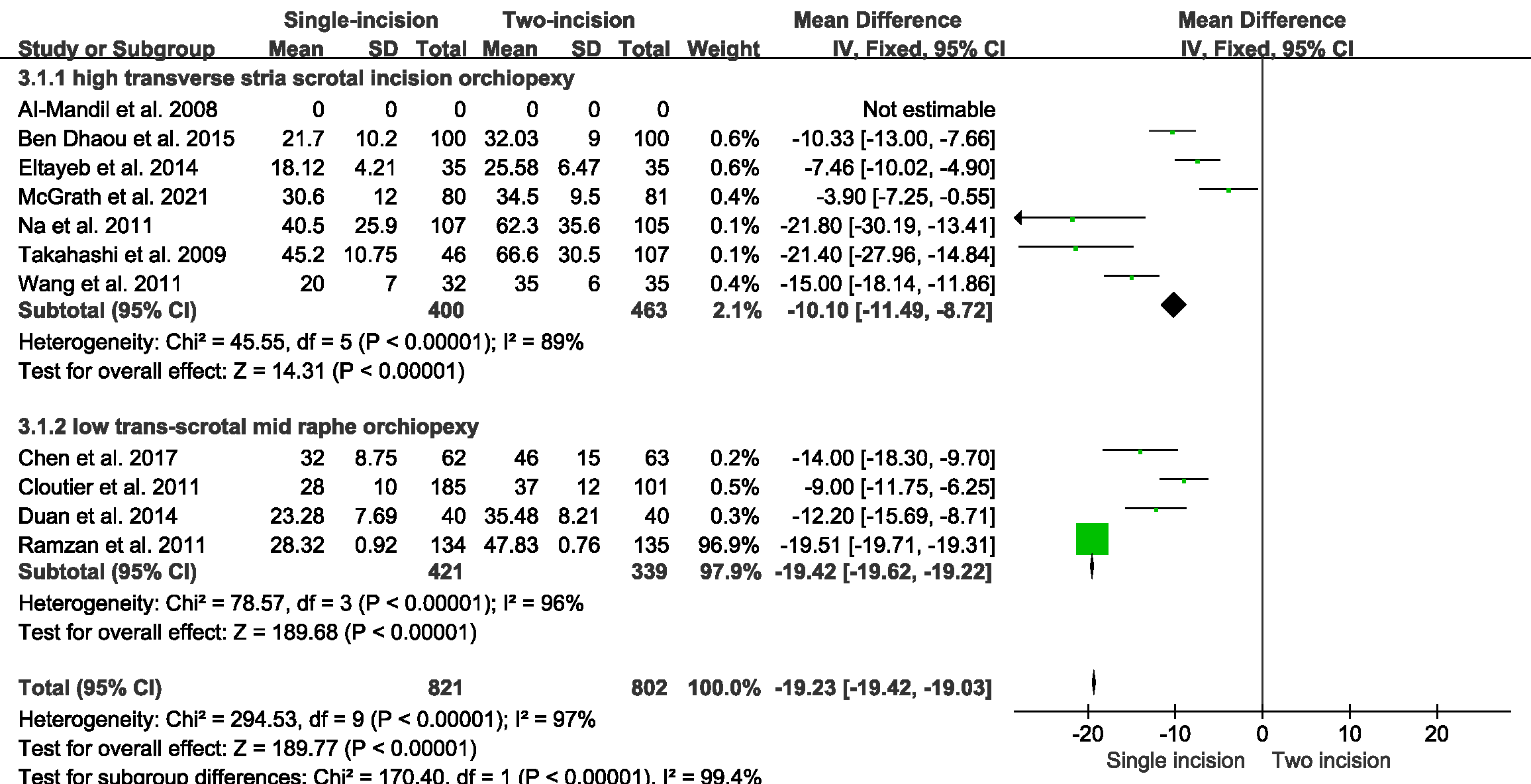

Supplement: Supplementary Figure 1 — The forest plot of comparison of operation time between high transverse stria single-incision scrotal orchiopexy and low trans-scrotal mid raphe single-incision orchiopexy and traditional two-incision inguinal orchiopexy. [file Image_1.TIF]

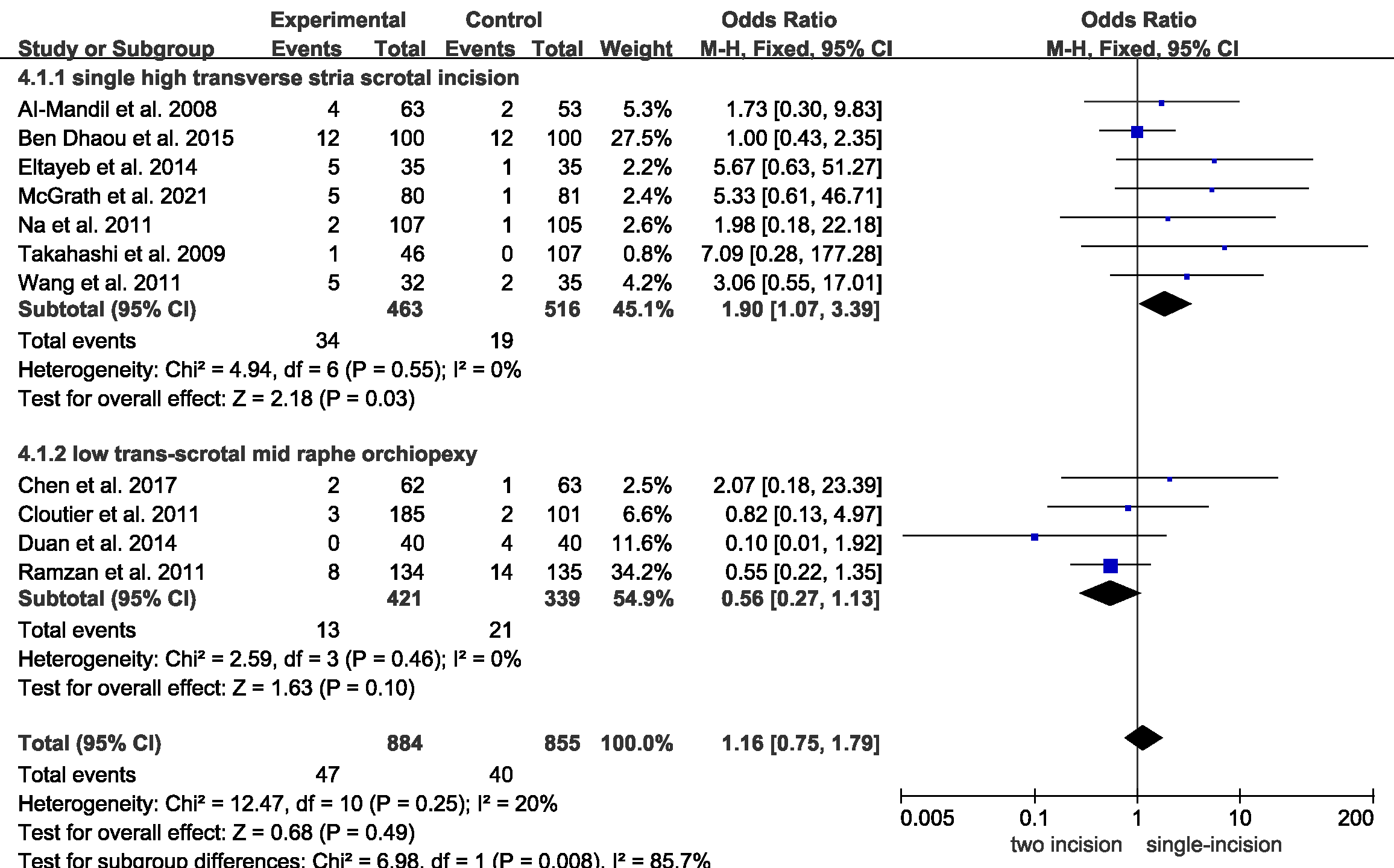

Supplement: Supplementary Figure 2 — The forest plot of comparison of total complications between high transverse stria single-incision scrotal orchiopexy and low trans-scrotal mid raphe single-incision orchiopexy and traditional two-incision inguinal orchiopexy. [file Image_2.TIF]
