# Supplementary material for: Comparison of Single-Incision Scrotal Orchiopexy and Traditional Two-Incision Inguinal Orchiopexy for Primary Palpable Undescended Testis in Children: A Systematic Review and Meta-Analysis
Source: Front Pediatr. 2022 Mar 15;10:805579. doi: 10.3389/fped.2022.805579 (PMC8964791; doi:10.3389/fped.2022.805579)
Supplement: Supplementary Table 1 — Quality assessment of included retrospective designed case control studies according to Newcastle–Ottawa Quality Assessment Scale (NOS), and randomized controlled trials according to Jadad Scale. [file Table_1.docx]

**Supplemental Table 1** Quality assessment of included retrospective designed case control studies according to Newcastle-Ottawa Quality Assessment Scale (NOS), and randomized controlled trials according to Jadad Scale

| **Quality assessment of retrospective designed case control studies** | | | | | | | | | |
| --- | --- | --- | --- | --- | --- | --- | --- | --- | --- |
| **Reference** | **Selection** | | | | **Comparability** | **Exposure** | | | **Scores** |
|  | Adequacy of case definition | Representativeness of cases | Selection of controls | Definition of controls | Comparability of cases and controls | Ascertainment of exposure | Same ascertainment for cases and controls | Non-response rate |  |
| AI-Mandil et al. 2008 [] | ***** | NA | NA | ***** | ***** | ***** | ***** | ***** | 6 |
| Chen et al. 2017 [] | ***** | NA | NA | ***** | ***** | NA | ***** | NA | 4 |
| Cloutier et al. 2011 [] | ***** | NA | NA | ***** | ***** | ***** | ***** | ***** | 6 |
| Cuda et al. 2011 [] | ***** | ***** | NA | ***** | ***** | ***** | ***** | NA | 6 |
| Duan et al. 2014 [] | ***** | NA | NA | ***** | ***** | NA | NA | ***** | 4 |
| Lee et al. 2009 [] | ***** | NA | NA | ***** | ***** | NA | NA | ***** | 4 |
| Sutton et al. 2011 [] | ***** | ***** | NA | ***** | ***** | NA | NA | ***** | 5 |
| Takahashi et al. 2009 [] | ***** | NA | NA | ***** | ***** | NA | ***** | ***** | 5 |
| Wang et al. 2011 [] | ***** | NA | NA | ***** | ***** | NA | NA | ***** | 4 |
| Yi et al. 2013 [] | ***** | NA | NA | ***** | ***** | NA | ***** | ***** | 5 |
| **Quality assessment of prospective designed randomized controlled studies** | | | | | | | | | |
| **Reference** | **Randomization** | | | | **Blinding** | **Withdrawals** | | | **Scores** |
| Badbarin et al. 2019 [] | 2, random numbers were produced by RandList 1.2 software | | | | 0, no blinding method described | 1, detailed description about withdraws or lost to follow-up | | | 3 |
| Ben Dhaou et al. 2015 [] | 1, mentioned random allocation while no description about random sequence | | | | 0, no blinding method described | 1, all patients were followed | | | 2 |
| Eltayeb et al. 2014 [] | 1, mentioned random allocation while no description about random sequence | | | | 0, no blinding method described | 1, all patients were followed | | | 2 |
| McGrath et al. 2021 [] | 2, computer generated web-based randomization sequence | | | | 2, doble-blinding and well described | 1, detailed description about withdraws or lost to follow-up and reasons | | | 5 |
| Na et al. 2011 [] | 1, mentioned random allocation while no description about random sequence | | | | 0, no blinding method described | 1, detailed description about withdraws or lost to follow-up and reasons | | | 2 |
| Nazem et al. 2019 [] | 1, mentioned random allocation while no description about random sequence | | | | 0, single-blinding | 1, detailed description about withdraws or lost to follow-up and reasons | | | 2 |
| Ramzan et al. 2011 [] | 1, mentioned random allocation while no description about random sequence | | | | 0, no blinding method described | 1, all patients were followed | | | 2 |
